# Involvement of FoxO1, Sp1, and Nrf2 in Upregulation of Negative Regulator of ROS by 15d-PGJ_2_ Attenuates H_2_O_2_-Induced IL-6 Expression in Rat Brain Astrocytes

**DOI:** 10.1007/s12640-020-00318-6

**Published:** 2022-01-08

**Authors:** Chen-Yu Wang, Chien-Chung Yang, Li-Der Hsiao, Chuen-Mao Yang

**Affiliations:** 1 Department of Pharmacology, College of Medicine, China Medical University, Taichung, 40402 Taiwan; 2 Department of Traditional Chinese Medicine, Chang Gung Memorial Hospital At Tao-Yuan, Kwei-San, Tao-Yuan, 33302 Taiwan; 3 School of Traditional Chinese Medicine, College of Medicine, Chang Gung University, Kwei-San, Tao-Yuan, 33302 Taiwan; 4 Ph.D. Program for Biotch Pharmaceutical Industry, China Medical University, Taichung, 40402 Taiwan; 5 Department of Post-Baccalaureate Veterinary Medicine, College of Medical and Health Science, Asia University, Wufeng, Taichung, 41354 Taiwan

**Keywords:** Hydrogen peroxide, IL-6, NADPH oxidase, NRROS, p47^phox^, 15d-PGJ_2_

## Abstract

Excessive production of reactive oxygen species (ROS) by NADPH oxidase (Nox) resulted in inflammation. The negative regulator of ROS (NRROS) dampens ROS generation during inflammatory responses. 15-Deoxy-∆12,14-prostaglandin J_2_ (15d-PGJ_2_) exhibits neuroprotective effects on central nervous system (CNS). However, whether 15d-PGJ_2_-induced NRROS expression was unknown in rat brain astrocytes (RBA-1). NRROS expression was determined by Western blot, RT/real-time PCR, and promoter activity assays. The signaling components were investigated using pharmacological inhibitors or specific siRNAs. The interaction between transcription factors and the NRROS promoter was investigated by chromatin immunoprecipitation assay. Upregulation of NRROS on the hydrogen peroxide (H_2_O_2_)-mediated ROS generation and interleukin 6 (IL-6) secretion was measured. 15d-PGJ_2_-induced NRROS expression was mediated through PI3K/Akt-dependent activation of Sp1 and FoxO1 and established the essential promoter regions. We demonstrated that 15d-PGJ_2_ activated PI3K/Akt and following by cooperation between phosphorylated nuclear FoxO1 and Sp1 to initiate the NRROS transcription. In addition, Nrf2 played a key role in NRROS expression induced by 15d-PGJ_2_ which was mediated through its phosphorylation. Finally, the NRROS stable clones attenuated the H_2_O_2_-induced ROS generation and expression of IL-6 through suppressing the Nox-2 activity. These results suggested that 15d-PGJ_2_-induced NRROS expression is mediated through a PI3K/Akt-dependent FoxO1 and Sp1 phosphorylation, and Nrf2 cascade, which suppresses ROS generation through attenuating the p47^phox^ phosphorylation and gp91^phox^ formation and IL-6 expression in RBA-1 cells. These results confirmed the mechanisms underlying 15d-PGJ_2_-induced NRROS expression which might be a potential strategy for prevention and management of brain inflammatory and neurodegenerative diseases.

## Introduction

Astrocytes exert several functions including production of growth factors and participating in the immune and repairing responses to brain injuries (Blackburn et al. 2009). In homeostatic maintenance, astrocytes interact with neurons to facilitate ROS detoxification during oxidative insults (Shih et al. 2003) which are involved in neurodegenerative diseases (Ricci et al. 2009). Importantly, astrocytes exert neuroprotection such as uptake of excitotoxic glutamate, protecting against oxidative stress, and limiting the spread of inflammatory cells or infectious agents (Sofroniew and Vinters 2010). Our previous studies indicate that excessive ROS generation in activated astrocytes induces the expression of inflammatory mediators resulting in neuronal apoptosis (Yang et al. 2013).

Excessive ROS could impair cellular functions and enhance inflammatory responses through the expression of inflammatory genes via various signaling pathways (Finkel 2003) in the pathogenesis of human diseases (Chrissobolis and Faraci 2008). The imbalance between ROS generation and antioxidants results in several pathologies of degenerative diseases (Lin and Beal 2006; Poljsak et al. 2013). To protect against the external stresses, ROS are essential for many physiological functions including the killing of invading microorganisms (Chrissobolis and Faraci 2008). The NADPH oxidase (Nox) complex is a major source of ROS in several physiological and pathological processes (Lee and Yang 2012; Rahman et al. 2006). The levels of Nox-dependent ROS generation contribute to the expression of either inflammatory or anti-inflammatory mediators. External stimuli trigger the signal components to initiate the activity of the Nox complex (Lee and Yang 2012). However, the negative regulatory mechanism of Nox activity is still unclear. Until Noubade et al. (2014) identified a novel regulatory gene, negative regulator of ROS (NRROS), limits ROS generation during inflammatory responses. The up-regulation of NRROS interferes with the association between Nox-2 and p22^phox^, followed by Nox-2 degradation and impediment of the Nox complex formation (Noubade et al. 2014). The zymosan-induced ROS generation is reduced by the expression of NRROS in IFN-γ-primed bone marrow-derived macrophages. Deficiency of NRROS gene shows more susceptible to microglial development and neurological disorders (Wong et al. 2017). These results suggested that NRROS could protect against neuroinflammatory disorders. In addition, NRROS has been shown to inhibit the NF-κB activity through interrupting the function of Toll-like receptors (Kim et al. 2015; Liu et al. 2013). Although the protecting effects of NRROS have been studied, the detail mechanisms of NRROS expression are still unknown in rat brain astrocytes (RBA-1).

15d-PGJ_2_ plays protecting effects in diverse cell systems (Abdo et al. 2012; Lin et al. 2006) and suppresses the p22^phox^ expression to protect against apoptosis of neurons and brain endothelial cells (Wu et al. 2016, 2014). Moreover, 15d-PGJ_2_ exerts neuroprotection from oxidative stress in astrocytes (Haskew-Layton et al. 2013). These protecting effects of 15d-PGJ_2_ are mediated through upregulation of Nrf2-dependent antioxidant proteins (Itoh et al. 2004). Therefore, 15d-PGJ_2_ might induce NRROS expression and attenuate the ROS-dependent inflammatory responses in RBA-1. We found that 15d-PGJ_2_ stimulated FoxO1 phosphorylation through PI3K/Akt pathway and activated Sp1 to regulate the NRROS transcription and Nrf2. The constitutive expression of NRROS attenuated the p47^phox^ phosphorylation to suppress ROS generation and IL-6 expression induced by H_2_O_2_. Thus, the up-regulation of NRROS by 15d-PGJ_2_ could protect against brain inflammatory responses.

## Materials and Methods

### Antibodies and Chemicals

DMEM/F-12 medium, FBS, TRIzol reagent, CM-H2DCFDA, and PLUS-Lipofectamine were from Invitrogen (Carlsbad, CA, USA). Hybond C membrane and enhanced chemiluminescence (ECL) detection system were from GE Healthcare Biosciences (Buckinghamshire, UK). NRROS (AAS08559C) antibody was from Antibody Verify (Las Vegas, NV, USA). Akt (rabbit polyclonal antibody, Cat# sc-8312, RRID:AB_671714), Lamin A (rabbit polyclonal antibody, Cat# sc-20680, RRID:AB_648148), Sp1 (rabbit polyclonal antibody, Cat# sc-14027, RRID:AB_2171049), and p22 (rabbit polyclonal antibody, Cat# sc-20781, RRID:AB_2090309) antibodies were from Santa Cruz (Santa Cruz, CA, USA). FoxO1 (rabbit monoclonal antibody, Cat# ab52857, RRID:AB_869817), Nox2 (rabbit monoclonal antibody, Cat# ab129068, RRID:AB_11144496), p85 (rabbit monoclonal antibody, Cat# ab40755, RRID:AB_777258), DDDDK tag (rabbit polyclonal antibody, Cat# ab1162, RRID:AB_298215), phospho-Sp1 (rabbit polyclonal antibody, Cat# ab37707, RRID:AB_1524434), Nrf2 (mouse monoclonal antibody, Cat# ab89443, RRID:AB_2041334), and phospho-Nrf2 (phospho-Ser40, Rabbit monoclonal antibody, Cat# ab76026, RRID:AB_1524049) antibodies were from Abcam (Cambridge, UK). Phospho-Akt (rabbit polyclonal antibody, Cat# 9271, RRID:AB_329825) and phospho-FoxO1 (rabbit polyclonal antibody, Cat# 9461, RRID:AB_329831, phospho-S256 FoxO1) antibodies were from Cell Signaling (Danvers, MA, USA). Anti-phospho-p47 (rabbit polyclonal antibody, Cat# A1171, RRID:AB_10696129) antibody was from Assay Biotech (Sunnyvale, CA, USA). Anti-glyceraldehyde 3-phosphate dehydrogenase (GAPDH) (Cat# MCA-1D4, RRID:AB_2107599) antibody was from EnCor Biotechnology (Gainesville, FL, USA). 15d-PGJ_2_ and 15d-PGJ_2_ antibody (Cat# ADI-915-043-100) were from Enzo (New York, NY, USA). AS1842856 was from Millipore (Billerica, MA, USA). AKT inhibitor VIII was from Cayman (Ann Arbor, MI, USA). SDS-PAGE reagents were from MDBio Inc (Taipei, Taiwan). Actinomycin D, LY294002, mithramycin A, and other chemicals were from Sigma (St. Louis, MO, USA).

### Cell Culture and Treatment

Cells from a rat brain astrocytic cell line (RBA-1) were used in this study. The maintenance and subculture of RBA-1 cells were performed as previously described (Lin et al. 2014). The use of the cell lines had been approved by Chang Gung University Institutional Animal Care and Use Committee (IACUC Approval No. CGU16-081). In brief, a primary astrocyte culture was isolated from neonatal rat cerebrum and the cell line was established naturally through successive cell passages. RBA-1 cells were identified with glial fibrillary acid protein (GFAP, an astrocyte-specific marker) staining and exhibited over 95% positive staining cells. Cells were plated onto culture plates and made quiescent at confluence by incubation in serum-free DMEM/F-12 for 24 h and then treated with 15d-PGJ_2_ at 37 °C for the indicated time intervals. When the inhibitors were used, cells were pretreated with the individual inhibitor for 1 h before exposure to 15d-PGJ_2_. The cytotoxicity of 15d-PGJ_2_ and pharmacological inhibitors at the incubation time was checked using an XTT assay kit, showing no significant effect on cell viability. Experiments were performed with cells from passages 7 to 12. No institutional ethical approval was required.

### Preparation of Cell Extracts, Cellular Fraction Extracts, and Western Blot Analysis

After treatment with 15d-PGJ_2_, RBA-1 cells were washed with ice-cold PBS, scraped, and collected with SDS-loading buffer (0.1 M Tris–HCl, pH 6.8; 1% SDS; 5% glycerol; 2.5% β-mercaptoethanol; 0.02% bromophenol blue). The nuclear and cytoplasmic fractions were isolated according to the protocol of NE-PER nuclear and cytoplasmic extraction reagents (ThermoFisher, Waltham, MA, USA). Samples were denatured, separated on SDS-PAGE, transferred to nitrocellulose membranes, and then probed with a respective primary antibody overnight at 4 °C. The washed membranes were incubated with an anti-rabbit horseradish peroxidase antibody (1:2000) for 1 h. The immunoreactive bands were detected using ECL reagents and captured by a UVP BioSpectrum 500 Imaging System (Upland, CA, USA). UN-SCAN-IT gel software (Orem, UT, USA) was used to analyze and quantify the image densitometry.

### Total RNA Extraction, Real Time-PCR, and PCR Analysis

RBA-1 cells were seeded on 10-cm culture dishes and treated with 15d-PGJ_2_. Total RNA was extracted with TRIzol reagent (Thermo Fisher, Waltham, MA, USA) according to the protocol of the manufacturer. The cDNA obtained from 5 μg total RNA was used to be a template for real-time PCR amplification. Real-time PCR was performed with KAPA PROBE FAST ABI Prism® qPCR kit (KK4705, Kapa Biosystems, Wilmington, MA, USA) and 7500 Real-Time PCR System (Applied Biosystems, Foster City, CA, USA) using the sequences of primers as follows:

Rat NRROS:

Forward primer: 5′-CTCATGCTTCAGAACCTCTCCTG-3′

Reverse primer: 5′-CAGAGTCGTCGAGCCTCATG-3′

Probe: 5′-TGGAGGTCGTGTCCTTGGCAAGAA-3′

Rat IL-6:

Forward primer: 5′-CGAAAGTCAACTCCATCTGCC-3′

Reverse primer: 5′-GGCAACTGGCTGGAAGTCTCT-3′

Probe: 5′-TCAGGAACAGCTATGAAGTTTCTCTCCG-3′

Human NRROS:

Forward primer: 5′-TTTTCACTTCCTGACCGTGG-3′

Reverse primer: 5′-CACCAACTTGCAGACTCCTTG-3′

Probe: 5′-AGGAACAGAAGCGGAACAGCCACA-3′

GAPDH:

Forward primer: 5′-AACTTTGGCATCGTGGAAGG-3′

Reverse primer: 5′-GTGGATGCAGGGATGATGTTC-3′

Probe: 5′-TGACCACAGTCCATGCCATCACTGC-3′

Relative gene expression was determined by the ΔΔ^Ct^ method, where Ct meant the threshold cycle. All experiments were performed in triplicate.

### Plasmid Constructions, Transfection, and Luciferase Reporter Gene Assays

To study the promoter activity, we constructed the luciferase reporter plasmids to investigate the promoter regulation in RBA-1 cells. The rat NRROS promoter plasmids were inserted into an pGL3-basic vector, respectively. The DNA fragments, rat NRROS promoter regions from − 1716 to + 135 bp, deletion fragments and human NRROS promoter from − 1053 to + 406, were constructed into an pGL3-basic vector. Luciferase plasmid and pCMV-β-gal were co-transfected into RBA-1 cells with Lipofectamine 2000. Promoter activity was detected using a luciferase assay system (Promega, Madison, WI, USA) to analyze the firefly luciferase activities and standardized with β-galactosidase activity. Site-directed mutagenesis of human FoxO1, a Ser256-to-Asp FoxO1 mutant (FoxO1^S256D^) mutant was constructed by inserting the DNA fragments encoding human FoxO1^S256D^ between the *Eco*RV and *Hind* III sites of pCMV-Tag2B (Stratagene, Santa Clara, CA, USA).

### Transient Transfection with siRNAs

Quiescent RBA-1 (80% confluence) were washed once with PBS and added 2 ml of serum-free DMEM/F-12 medium to each well (6-well plate). The siRNAs of scrambled, AKT1 (RSS301983, 5′-UUAGGAUGAGCUCGAACAGCUUCUC-3′), and p85 (Pik3r1, RSS303756) were obtained from Thermo Fisher (Waltham, MA, USA); the siRNAs of Sp1 (Rn01_00038010, 5′-CAUUAUUGCUGCUAUGCCA-3′) and FoxO1 (Rn02_00284211, 5′-CUAUUCAUUUGCACCGCCA-3′) were obtained from Sigma (St. Louis, MO, USA). Nrf2 (nfe212-RSS343557) was from Invitrogen (Carlsbad, CA, USA). Transient transfection siRNAs (100 nM) was performed using a GeneMute reagent according to the manufacturer’s instructions (Rockville, MD, USA).

### Chromatin Immunoprecipitation Assay

Chromatin immunoprecipitation (ChIP) was performed as previously described with minor modifications (Chi et al. 2011). In brief, RBA-1 cells were fixed with 1% formaldehyde at room temperature for 30 min and then stopped the cross-linkage reaction with 0.125 M glycine. The cells were washed with cold-PBS and harvested with 500 μl ChIP lysis buffer. The fixed chromatins were broken down with sonication to about 500 to 100 bp. The equal concentrations of cell lysates were incubated with 2 μg respective antibody and rotated at 4 °C for 1 h. Twenty microliters ChIP beads (Millipore) was added to the mixture and rotated at 4 °C overnight. After washing, the immunocomplex was eluted with elution buffer and de-cross-linkage at 65 ℃ for overnight. The genomic DNA fragment was extracted from the antibody-absorbed complex. PCR reactions were performed by using response element-specific primers and analyzed with 2% agarose gel electrophoresis.

### Stable Clone Construction and Establishment of Human NRROS in RBA-1 Cells

The DNA fragment of the human NRROS amino acid encoding region was inserted between BamHI and HindIII sites of pCMV-Tag2B to construct the expression plasmid, pCMV-Tag2B-hNRROS. RBA-1 cells (about 70% confluence) were plated on a 10-cm dish and transfected with 2 μg of pCMV-Tag2B-hNRROS or pCMV-Tag2B using Xtreme® DNA transfection reagent. Twenty-four hours after transfection, cells were split and selected by cultivated in G418 (500 μg/ml)-containing medium with 5% CO_2_ at 37 °C. Cells were then sub-cultured every 3 days for 2 weeks to obtain stable clones by collecting drug-resistant colonies. The level of human NRROS expression of the RBA-1 stable clone was analyzed by Western blot and probed with a mouse anti-Flag antibody.

### ROS Generation Assay

The levels of ROS were detected using CM-H_2_DCFDA (C6827, Invitrogen) as previously described (Lee et al. 2013). Briefly, cells were washed twice with warmed serum-free medium and then incubated with CM-H_2_DCFDA at 37 ℃ for 30 min and then treated with 100 μM H_2_O_2_ for 2 h. The cells were washed twice with warm PBS and observed under a fluorescent microscope.

### IL-6 ELISA

Cells were treated with 100 μM H_2_O_2_ for 2 h. The medium was harvested to analyze the level of IL-6 release using an IL-6 enzyme-linked immunosorbent assay (ELISA) kit (Biolegend, San Diego, CA, USA).

### Data and Statistical Analysis

Statistical analysis was performed by using GraphPad Prism Program 7.0 software (RRID:SCR_000306, GraphPad, San Diego, CA, USA). We used one-way ANOVA followed by Dunnett’s post hoc test when comparing more than two groups of data and one-way ANOVA, nonparametric Kruskal–Wallis test, followed by Dunnett’s post hoc test when comparing multiple independent groups. *P* values of 0.05 were considered to be statistically significant. Post hoc tests were run only if *F* achieved *P* < 0.05, and there was no significant in homogeneity of variance. No sample calculation was performed. We did not conduct any data test for outliers, and thus, no single data points were excluded. All the data were expressed as the mean ± SEM, at least three individual experiments (*n* = number of independent cell culture preparations). The *n* values are provided in the figure legends. Error bars were omitted when they fell within the dimensions of the symbols.

## Results

### 15d-PGJ_2_ Induces NRROS Expression

**Fig. 1:**
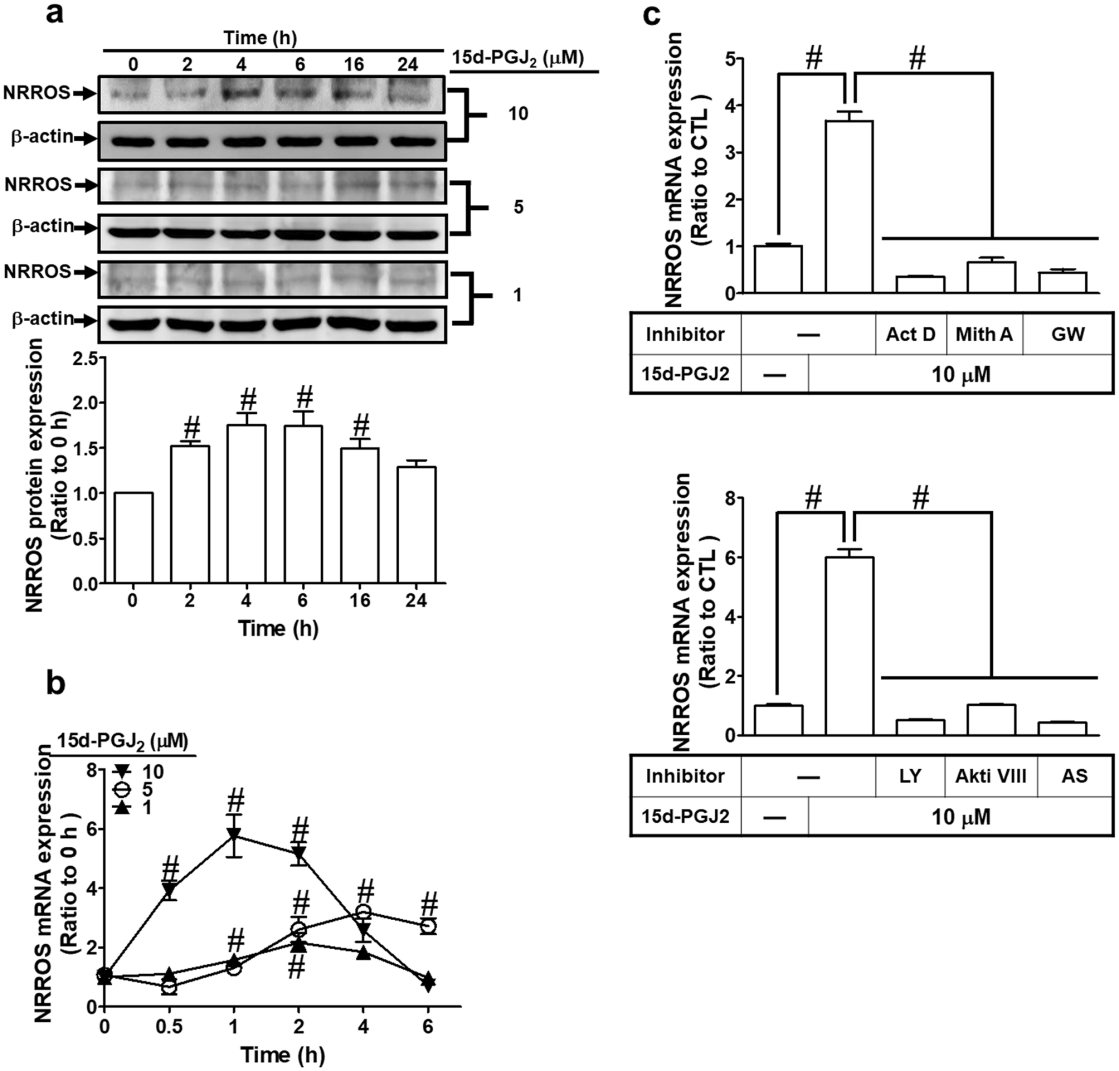
15d-PGJ_2_ induced NRROS expression in RBA-1 cells. **a**, **b** RBA-1 cells were treated with 1, 5, or 10 μM 15d-PGJ_2_ for the indicated time intervals. The levels of **a** NRROS protein and **b** mRNA expression were analyzed by Western blot and RT/real-time PCR, respectively. Lower panel of **a** indicates the quantitation of 10 μM 15d-PGJ_2_ treatment. **c** RBA-1 cells were pretreated with actinomycin D (ActD, 1 μM), mithramycin A (Mith A, 1 μM), GW9662 (GW, 10 nM), LY294002 (10 μM), Akti VIII (1 μM), or AS1842856 (AS, 0.3 μM) for 1 h and then treated with 10 μM 15d-PGJ_2_ for 2 h. The levels of NRROS mRNA expression were analyzed by RT/real-time PCR. The data are expressed as mean ± SEM, from three independent experiments (*n* = 3, number of independent cell culture preparations). ^#^*p* < 0.05, as compared with the respective values significant difference as indicated

NRROS exerts a negatively regulatory effect on ROS generation (Noubade et al. 2014). 15d-PGJ_2_ has been shown to display neuroprotection against ROS stress (Abdo et al. 2012; Lin et al. 2006). The relationship between 15d-PGJ_2_ and expression of NRROS is still unknown. Here, we found that 15d-PGJ_2_ concentration- and time-dependently induced NRROS protein and mRNA expression in RBA-1 cells (*p* < 0.05, as compared with the control, Fig. 1a, b). Quantification of the Western blotting of 10 μM 15d-PGJ_2_, we found that NRROS protein was induced with a maximal response within 4–6 h (*p* < 0.05, as compared with the control) and slightly declined at 24 h (Fig. 1a, lower panel). The levels of NRROS mRNA expression were increased to about sixfold within 1 h (*p* < 0.05, as compared with the control) and then declined to the basal level within 6 h (Fig. 1b). The lower concentrations (1 and 5 μM) of 15d-PGJ_2_ induced a little of NRROS expression. Pretreatment with the inhibitor of transcription actinomycin D (Act. D, 1 μM), PPAR-γ (GW9662, 10 nM), Sp1 (mithramycin A, 1 μM), PI3K (LY294002, 10 μM), Akt inhibitor VIII (Akti VIII, 1 μM), or FoxO1 (AS1842856, 0.3 μM) attenuated the 15d-PGJ_2_-induced NRROS mRNA expression (*p* < 0.05, as compared with the control, Fig. 1c). These results suggested that 15d-PGJ_2_-induced transcriptional NRROS mRNA expression is, at least, mediated through PI3K/Akt, Sp1, FoxO1, and PPARγ in RBA-1 cells.

### Roles of Rat NRROS Promoter and 15d-PGJ_2_ Response Regions in NRROS Expression

**Fig. 2:**
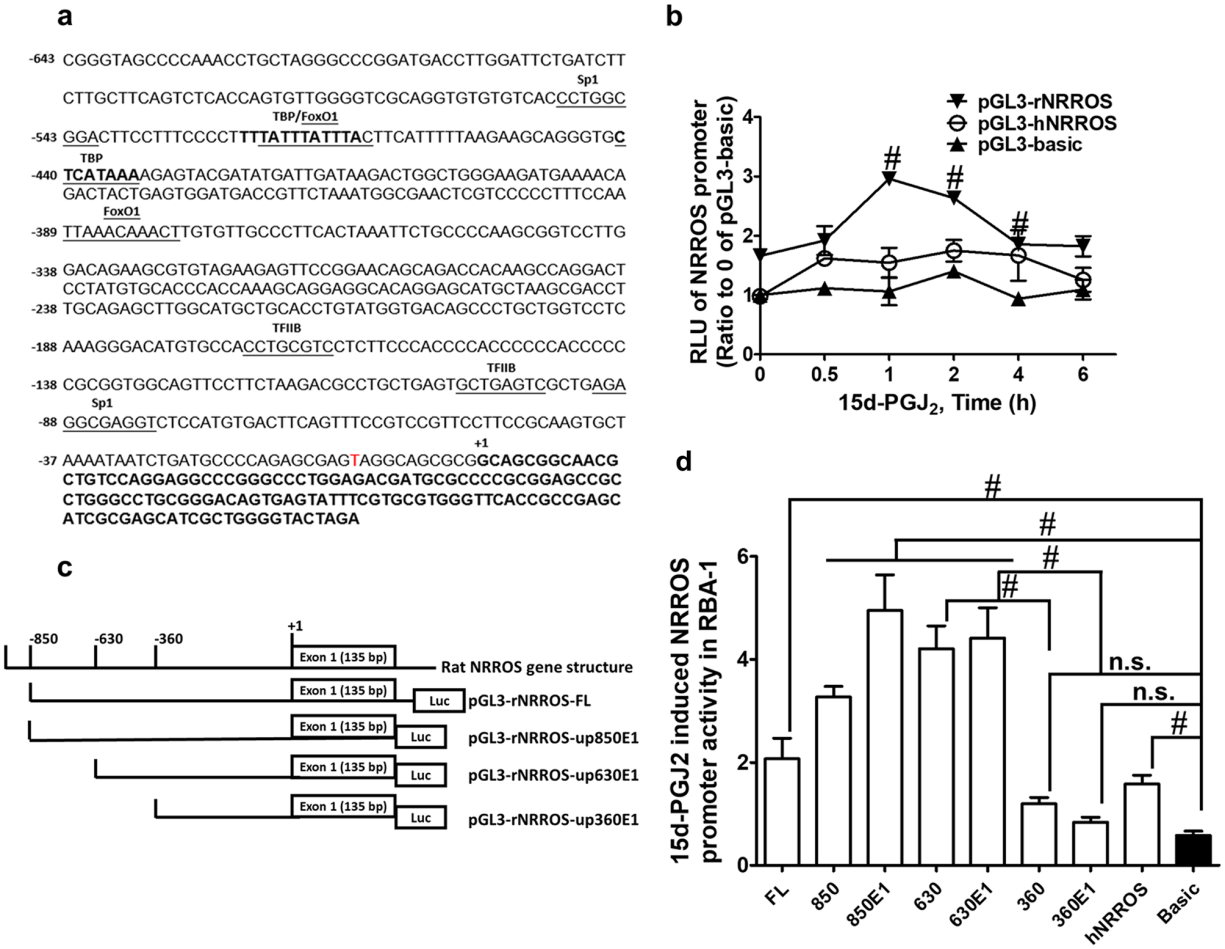
Analysis of rat NRTROS promoter and screening for 15d-PGJ_2_ response elements.** a** The DNA sequences of rat NRROS promoter and transcription factor binding sites. The exon 1 regions are shown in boldface and (+ 1) indicates the first nucleotide of the mRNA. **b** RBA-1 cells were transfected with either rat (r)NRROS or (h)NRROS plasmid, and pCMV-β-gal DNA for 24 h, and then treated with 10 μM 15d-PGJ_2_ for the indicated time intervals. The promoter activity was determined in the cell lysates using a promoter assay kit. **c** Scheme of the rat NRROS promoter region and reporter constructs. The position of deletion mutant, transcription initiation (+ 1), and location of exon 1 are indicated. Figures were not drawn to scale. **d** RBA-1 cells transfected with various NRROS constructs were treated with 10 μM 15d-PGJ_2_ for 1 h. The pGL3B-Luc without the promoter was used as a control (Basic). Deletion mutants were used to analyze the essential promoter regions and 15d-PGJ_2_ response element. RLU indicates the related luciferase units. Statistical analysis was determined using two-tailed unpaired Student’s *t* test. The data are presented as mean ± SEM, from three independent experiments (*n* = 3, number of independent cell culture preparations). ^#^*p* < 0.05, as compared with the respective values significantly different as indicated

To investigate the regulation of NRROS transcription, we analyzed the candidate sequences of NRROS promoter regions (upstream of transcription initiating site and the exon 1). Figure 2 a shows the sequences of rat NRROS upstream region and consensus motifs for transcription factor binding sites. There are several possibilities of response elements, such as Sp1, FoxO1, transcription factor IIB (TFIIB), and TATA-binding protein (TBP) on the promoter-predicted regions of NRROS. To analyze the DNA sequences of rat NRROS promoters, there was no typical location of TBP binding site (about 30–40 bp to initiation site) but several Sp1 binding sites on rat NRROS promoters. We speculated that NRROS belongs to the TATA-less promoters. Moreover, Sp1 was an important factor in NRROS mRNA expression which was attenuated by mithramycin A (Fig. 1c). To examine the transcriptional regulation, we constructed the luciferase reporter plasmids from rat NRROS promoters. The results of the promoter activity assay indicated that 15d-PGJ_2_ significantly induced rat NRROS promoter activity in RBA-1 cells (*p* < 0.05, as compared with the control, Fig. 2b). To investigate the location of 15d-PGJ_2_-response elements, serial plasmids of deletion mutants were constructed (Fig. 2c). These fragments of the NRROS promoter were transfected into RBA-1 cells to analyze the essential promoter regions for NRROS expression. As shown in Fig. 2d, up630E1 exhibited the maximal promoter activity (*p* < 0.05, as compared with the control), and the transcription activity of up360E1 was dramatically lower than that of the full-length construction (*p* < 0.05, as compared with the control, as compared with that of up630E1). These results suggested that the essential promoter region is located between the region of − 630 to − 360 bp to initiate NRROS transcription. Next, to examine the location of 15d-PGJ_2_-response element, these plasmid-transfected cells were treated with 15d-PGJ_2_ and analyzed the promoter activities. The results of the promoter activity assay indicated that plasmids of the full-length and − 850 bp obviously exhibited the 15d-PGJ_2_-induced promoter activities (Fig. 2d). These promoter analyses suggested that 360 bp upstream of transcription initiated site (starting site of exon 1) is the essential promoter region and the region between − 630 and − 360 contained the 15d-PGJ_2_ response elements for NRROS induction in RBA-1 cells.

### Involvement of PI3K/Akt and FoxO1 in 15d-PGJ_2_-Induced NRROS Expression

**Fig. 3:**
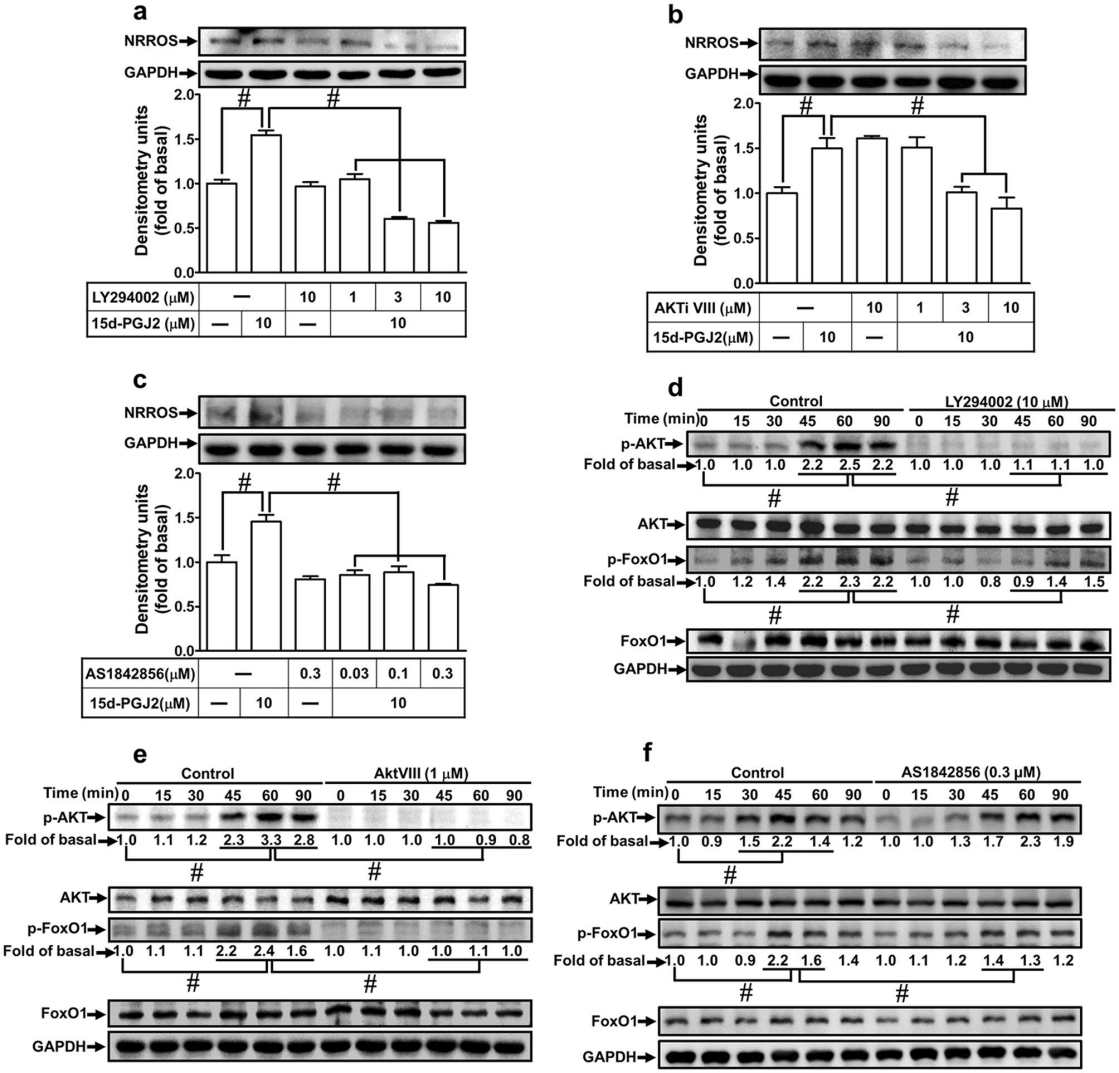
15d-PGJ_2_-induced NRROS expression is mediated through PI3K, AKT, and FoxO1. RBA-1 cells were pretreated with different concentrations of **a**, **d** LY294002, **b**, **e** AKTi VIII, or **c**, **f** AS1842856 for 1 h and then incubated with 10 μM 15d-PGJ_2_ for the indicated time intervals. The levels of NRROS, phospho-AKT, phospho-FoxO1, AKT, FoxO1, and GAPDH protein were analyzed by Western blotting. The data are presented as mean ± SEM, from three independent experiments (*n* = 3, number of independent cell culture preparations). ^#^*p* < 0.05, as compared with the respective values significantly different as indicated

PI3K consisting of a catalytic subunit (p110) and a regulatory subunit (p85) and its downstream component of Akt regulate the expression of several proteins in various types of cells (Lin et al. 2014; Liu et al. 2009). To determine the roles of PI3K/Akt in NRROS induction, pretreatment with the inhibitor of PI3K (LY294002) or Akt (Akti VIII) concentration-dependently attenuated the 15d-PGJ_2_-induced NRROS protein expression (*p* < 0.05, as compared with the cells treated with 15d-PGJ_2_, Fig. 3a, b). The phosphorylation of the FoxO proteins, a subgroup of the Forkhead family of transcription factors, is activated by PI3K/Akt pathway leading to the expression of target genes (Huang and Tindall 2007). To determine the role of FoxO1 in the 15d-PGJ_2_-induced NRROS protein expression mediated through PI3K/Akt, pretreatment with a FoxO1 inhibitor (AS1842856) concentration-dependently attenuated the 15d-PGJ_2_-induced NRROS protein expression (*p* < 0.05, as compared with the cells treated with 15d-PGJ_2_, Fig. 3c). In addition, the involvement of PI3K/Akt and FoxO1 in the 15d-PGJ_2_-induced responses was confirmed by determining the phosphorylation of Akt and FoxO1. As shown in Fig. 3d–f, 15d-PGJ_2_ time-dependently stimulated the phosphorylation of Akt with a maximal response within 60 min (*p* < 0.05, as compared the control), which was attenuated by either LY294002 or Akti VIII, but not by AS1842856 (*p* < 0.05, as compared with the cells treated with 15d-PGJ_2_). Moreover, 15d-PGJ_2_ stimulated FoxO1 phosphorylation with a maximal response within 45–60 min (*p* < 0.05, as compared the control), which was attenuated by LY294002, Akti VIII, or AS1842856 (*p* < 0.05, as compared with the cells treated with 15d-PGJ_2_), implying that FoxO1 is a downstream component of PI3K/Akt. These results suggested that 15d-PGJ_2_-induced NRROS expression is mediated through a PI3K/Akt-dependent FoxO1 cascade in RBA-1 cells.

**Fig. 4:**
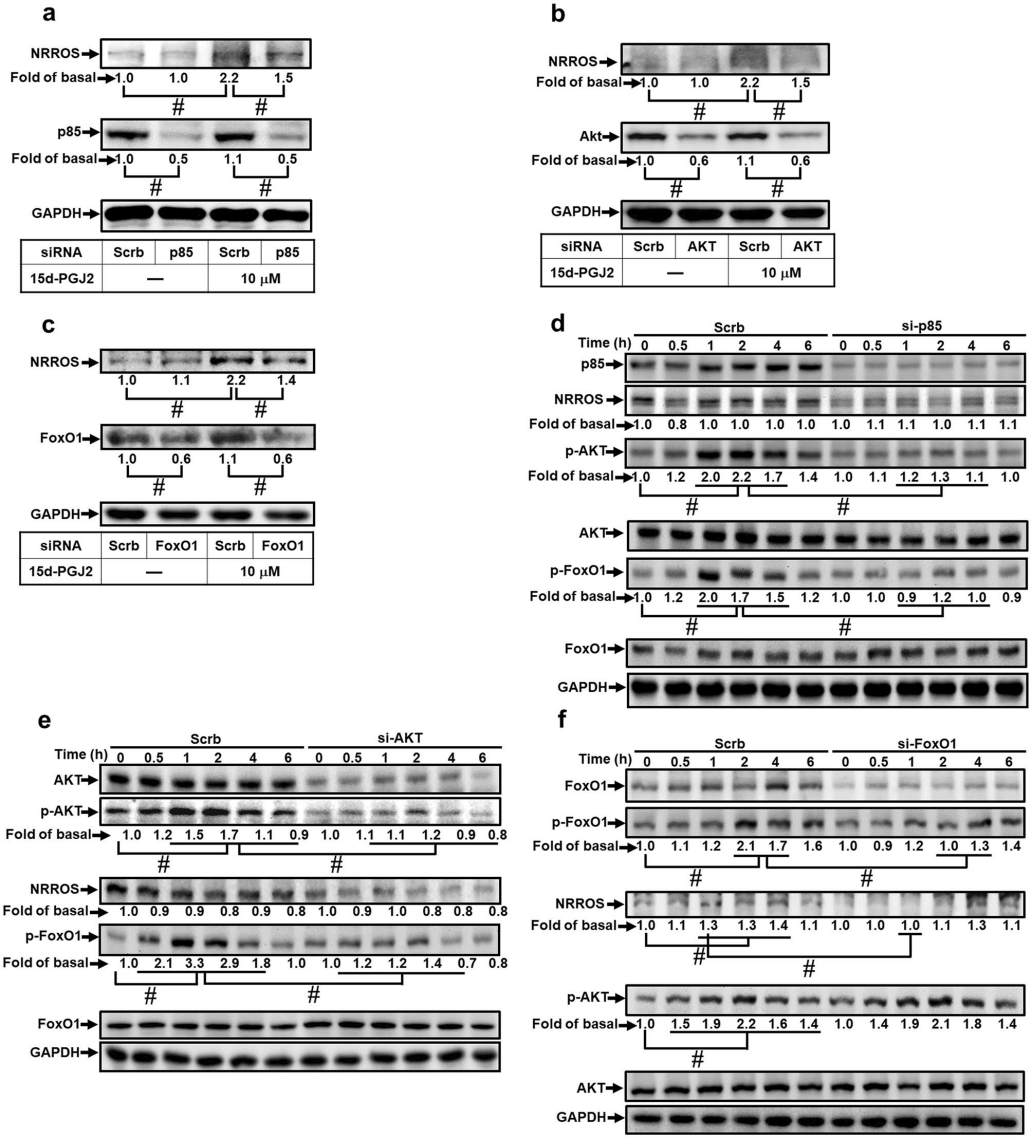
The roles of PI3K, AKT, and FoxO1 in 15d-PGJ_2_-induced NRROS expression are confirmed by transfection with respective siRNAs. RBA-1 cells were transfected with siRNA (**a**, **d** p85; **b**, **e **AKT, or **c**, **f** FoxO1) or scramble siRNA for 48 h and then incubated with 10 μM 15d-PGJ_2_ for 4 h (**a**–**c**) and for the indicated time intervals (**d**–**f**). The levels of NRROS, phospho-AKT, phospho-FoxO1, AKT, FoxO1, and GAPDH protein were analyzed by Western blotting. The data are presented as mean ± SEM, from three independent experiments (*n* = 3, number of independent cell culture preparations). ^#^*p* < 0.05, as compared with the respective values significantly different as indicated

To ensure the roles of PI3K/Akt and FoxO1 in NRROS expression, transfection with either p85 (Fig. 4a), Akt (Fig. 4b), or FoxO1 (Fig. 4c) siRNA reduced the protein level of p85 (by 55%), Akt (by 47%), or FoxO1 (by 39%), and then attenuated the 15d-PGJ_2_-induced NRROS protein expression (by 35%, 40%, or 41%, respectively). Moreover, 15d-PGJ_2_-stimulated Akt and FoxO1 phosphorylation were blocked by transfection with either p85 or Akt siRNA (*p* < 0.05, as compared with the cells treated with 15d-PGJ_2_, Fig. 4d, e). Knockdown of FoxO1 reduced the phosphorylation of FoxO1 only, but no effect on the phosphorylation of Akt (Fig. 4f). These results concluded that 15d-PGJ_2_-induced NRROS expression is mediated through a PI3K/Akt/FoxO1 pathway in RBA-1 cells.

### FoxO1 and Sp1 Involve in 15d-PGJ_2_-Induced NRROS Expression

**Fig. 5:**
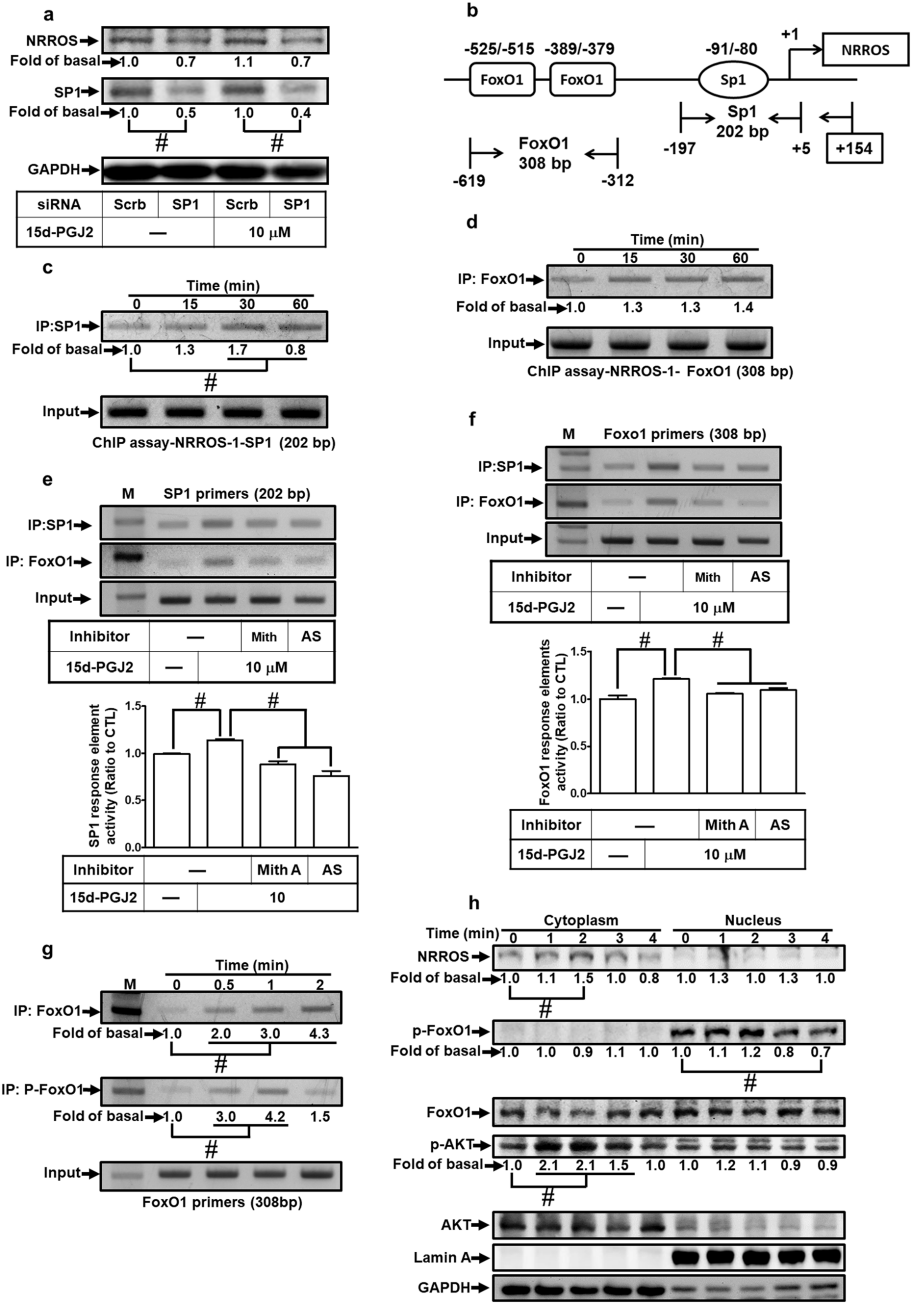
15d-PGJ_2_ stimulates the interaction between Sp1 and FoxO1 leading to NRROS expression.** a** RBA-1 cells were transfected with Sp1 siRNA and then incubated with 10 μM 15d-PGJ_2_ for 4 h. The levels of NRROS, Sp1, and GAPDH were analyzed by Western blotting. **b** Schematic representation of rat NRROS promoter. Locations of primers used for ChIP assays (Sp1 and FoxO1) and transcription factors associated regions are indicated. **c**, **d** ChIP assays were performed using an anti-Sp1 or anti-FoxO1 antibody and then amplified the DNA fragments with Sp1 and FoxO1 primers, respectively. **e**, **f** RBA-1 cells were pretreated without or with mithramycin A or AS1842856 for 1 h and then incubated with 15d-PGJ_2_ for 1 h. The association between FoxO1 and Sp1 on promoter was determined by ChIP assays. **g** Cells were treated with 10 μM 15d-PGJ_2_ for the indicated time intervals. ChIP assays were performed using an anti-FoxO1 or anti-phospho-FoxO1 antibody. **h** Cells were treated with 10 μM 15d-PGJ_2_ for the indicated time intervals. The levels of protein expression in either cytoplasmic or nuclear fractions were analyzed by Western blotting. The data are presented as mean ± SEM, from three independent experiments (*n* = 3, number of independent cell culture preparations). ^#^*p* < 0.05, as compared with the respective values significantly different as indicated

In the TATA-less promoter, Sp1 facilitates RNA polymerase II association on the promoter to initiate transcription (Smale and Kadonaga 2003). To ensure the role of Sp1 in the 15d-PGJ_2_-induced NRROS transcription, transfection with Sp1 siRNA knocked down the level of Sp1 protein (by 55%) and then attenuated the 15d-PGJ_2_-induced NRROS protein expression in RBA-1 cells (by 41%, *p* < 0.05, as compared with the cells treated with 15d-PGJ_2_, Fig. 5a). These results indicated that both Sp1 and FoxO1 contributes to regulate NRROS induction in the 15d-PGJ_2_-treated RBA-1 cells (Figs. 4c and 5a).

Based on our findings of rat NRROS promoter and 15d-PGJ_2_ induction (Fig. 2), we established that − 630 to − 360 is an essential promoter region and − 850 to − 630 region contained the 15d-PGJ_2_ response element in NRROS expression. To analyze these regions involved in NRROS expression, we found that the region of − 630 to − 360 contains several Sp1 binding sites and the region of − 850 to − 630 contains the FoxO1 response element. We speculated that both Sp1 and FoxO1 are involved in the 15d-PGJ_2_-mediated NRROS transcription. As shown in Fig. 5b, the schematic diagram represented the structure of rat NRROS promoter and transcription factors associated regions as indicated. To investigate the roles of these two transcription factors in NRROS expression, ChIP was performed to analyze the association between Sp1 and FoxO1 on rat NRROS promoter. The results indicated that 15d-PGJ_2_ stimulated the interaction between Sp1 and FoxO1 on NRROS promoter reaching a maximum within 30–60 min (*p* < 0.05, as compared the control, Fig. 5c, d), which was attenuated by either mithramycin A or AS1842856 (*p* < 0.05, as compared with the cells treated with 15d-PGJ_2_, Fig. 5e, f), respectively. These results strongly confirmed that both Sp1 and FoxO1 cooperatively regulate the 15d-PGJ_2_-induced NRROS transcription in RBA-1 cells.

### 15d-PGJ_2_-Induced Nuclear Accumulation and Phosphorylation of FoxO1 Associates with NRROS Promoter to Regulate NRROS Transcription

To examine the role of phosphorylated FoxO1 in the 15d-PGJ_2_-induced NRROS expression, ChIP assay was performed by using an anti-FoxO1 or anti-phospho-FoxO1 (Ser^256^) antibody to analyze the interaction between FoxO1 and rat NRROS promoters. We found that 15d-PGJ_2_ induced FoxO1 accumulation and phosphorylated FoxO1 binding to FoxO1 response element of NRROS promoters (*p* < 0.05, as compared the control, Fig. 5g). The 15d-PGJ_2_-stimulated phosphorylation of FoxO1 (Ser^256^) binding to the promoter was blocked by a FoxO1 inhibitor (AS1842856), but not by a Sp1 inhibitor (mithramycin A) (*p* < 0.05, as compared with the cells treated with 15d-PGJ_2_, Supplementary Fig. S1a) on the Sp1 binding region. On the FoxO1-binding regions, the results of ChIP indicated that both phosphorylated FoxO1 and Sp1 bindings were attenuated by AS1842856 or mithramycin A (*p* < 0.05, as compared with the cells treated with 15d-PGJ_2_, Supplementary Fig. S1b). Moreover, we determined the localization of NRROS protein in the subcellular fractions. As expected, NRROS accumulation in the cytoplasm occurred within 1–3 h of 15d-PGJ_2_ treatment (*p* < 0.05, as compared the control, Fig. 5h). Importantly, the levels of phospho-FoxO1 were obviously detected in the nuclear fraction (*p* < 0.05, as compared the level of cytoplasmic fraction, Fig. 5h). 15d-PGJ_2_ stimulated FoxO1 phosphorylation reaching a maximum within 1–2 h and declining at 3–4 h. We also evaluated the effects of phosphorylated FoxO1 on NRROS expression. Overexpression of either constitutively phosphorylated FoxO1 mutant (S256D-FoxO1) or dephosphorylated FoxO1 mutant (S256A-FoxO1) on RBA-1 cells was used to analyze the NRROS expression. We found that S256D-FoxO1 up-regulated NRROS expression and S256A-FoxO1 attenuated the 15d-PGJ_2_-induced NRROS expression (Supplementary Fig. S2). These findings indicated that the phosphorylated FoxO1 binding on NRROS promoters facilitates the transcriptional initiation in RBA-1 cells challenged with 15d-PGJ_2_.

### 15d-PGJ_2_-Stimulated Phosphorylation of Nrf2 Leading to NRROS Expression

**Fig. 6:**
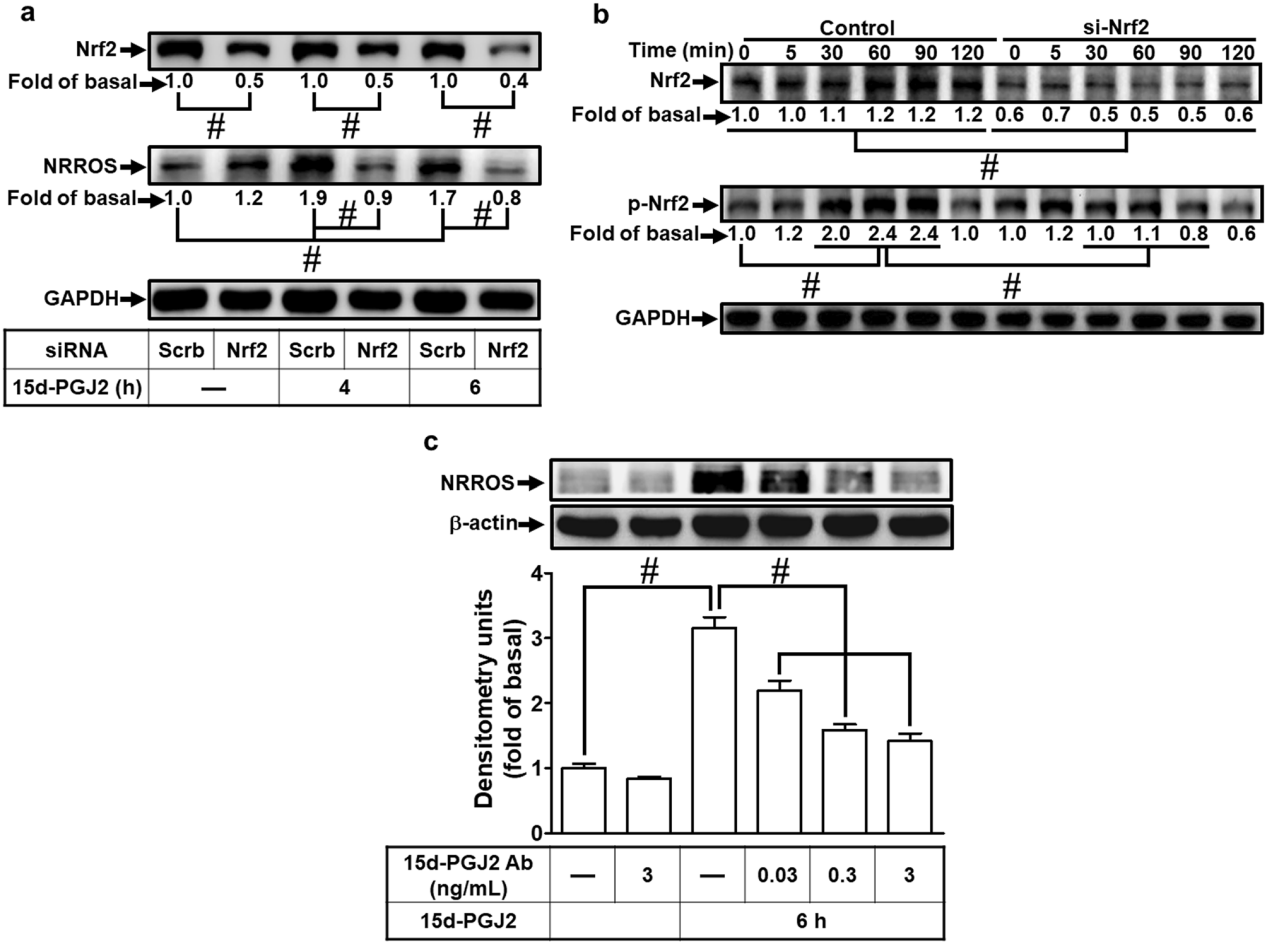
NRROS expression via Nrf2 activation by 15d-PGJ_2_ in RBA-1 cells. The cells were transfected with Nrf2 siRNA or scramble siRNA (**a**, **b**) for 48 h and then incubated with 10 μM 15d-PGJ_2_ for 4 h or 6 h (**a**), and indicated time intervals (**b**). **c** The cells were pretreated with various concentration of anti-15d-PGJ2 antibody for 1 h and then incubated with 10 μM 15d-PGJ_2_ for 6 h. The levels of NRROS, phospho-Nrf2, Nrf2, β-actin, and GAPDH protein were analyzed by Western blotting. The data are presented as mean ± SEM, from three independent experiments (*n* = 3, number of independent cell culture preparations). #*p* < 0.05, as compared with the respective values significantly different as indicated

15d-PGJ_2_ has been shown to induce the expression of antioxidant proteins in an Nrf2-dependent manner (Mochizuki et al. 2005). Thus, the role of Nrf2 in the expression of NRROS was investigated in RBA-1 cells challenged with 15d-PGJ_2_. We transfected the cells with Nrf2 siRNA which knocked down the levels of Nrf2 protein and attenuated the 15d-PGJ_2_-induced NRROS protein expression (Fig. 6a). Whether 15d-PGJ_2_ stimulated Nrf2 phosphorylation was determined in these cells. As shown in Fig. 6b, 15d-PGJ_2_ stimulated Nrf2 phosphorylation in a time-dependent manner, which was attenuated by transfection with Nrf2 siRNA. 15d-PGJ_2_ is an endogenous electrophile which could activate Nrf2 and induce the expression of antioxidant proteins (Mochizuki et al. 2005). Thus, the antibody against the 15d-PGJ_2_ was used to block its inductive effect on NRROS expression. RBA-1 cells were pretreated with 15d-PGJ_2_ antibody for 1 h and then incubated with 15d-PGJ_2_ for 6 h. We found that the antibody against the 15d-PGJ_2_ concentration-dependently attenuated NRROS expression (Fig. 6c).

## Overexpression of Human NRROS Reduces the H_2_O_2_-Induced ROS Generation and IL-6 Expression

**Fig. 7:**
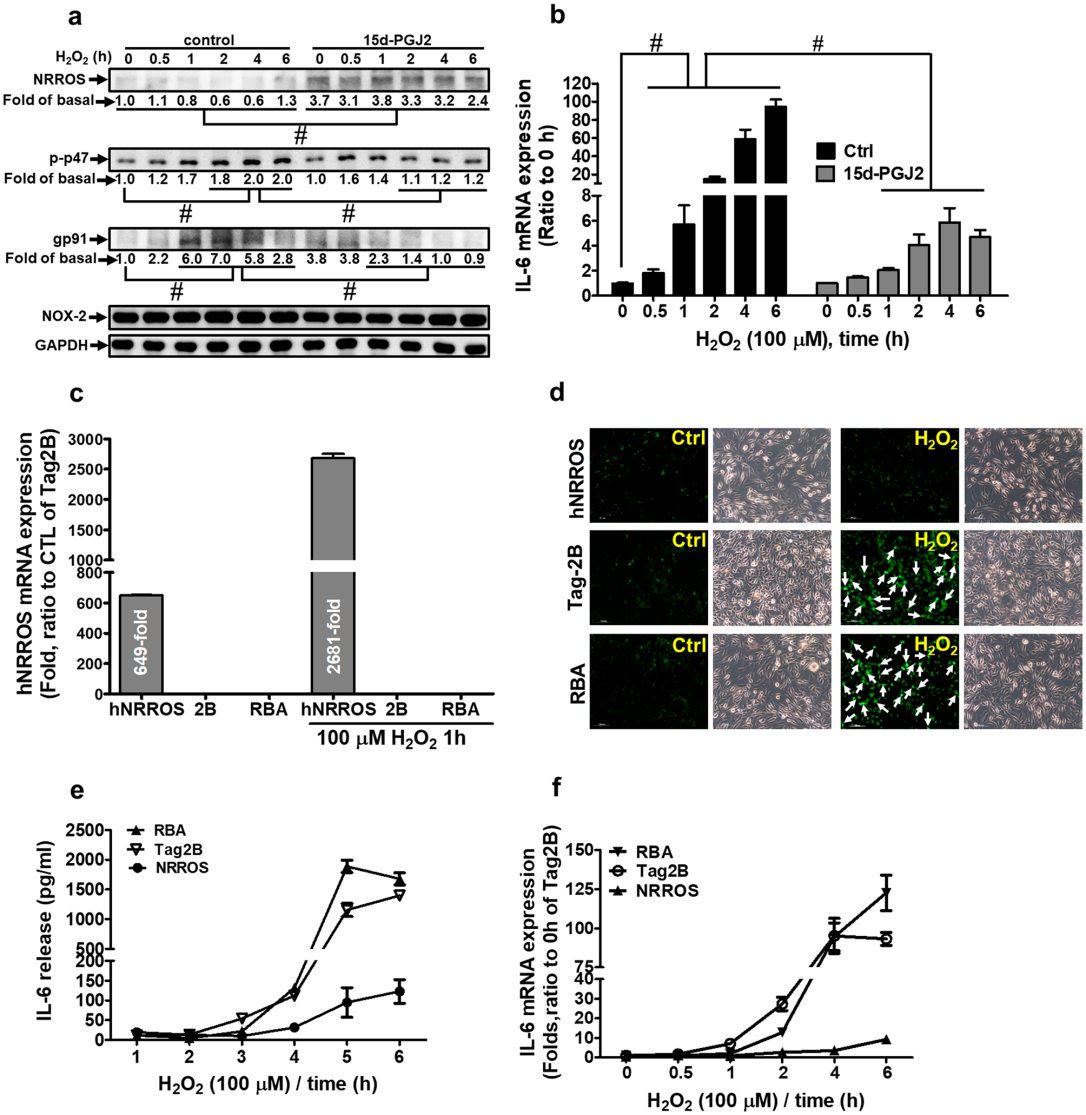
Overexpression of human NRROS reduces the H_2_O_2_-induced ROS generation, IL-6 expression, and p47 phosphorylation.** a** RBA-1 cells were pretreated with 10 μM 15d-PGJ_2_ for 1 h and then treated with 100 μM H_2_O_2_ for the indicated time intervals. The levels of protein expression were analyzed by Western blotting. **b** RBA-1 cells were pretreated with 15d-PGJ_2_ for 1 h and then treated with 100 μM H_2_O_2_ for the indicated time intervals. The levels of IL-6 mRNA expression were determined by RT/real-time PCR. **c**, **d** RBA-1 stable clones of hNRROS and pCMV-Tag2B, or RBA-1 cells were treated with 100 μM H_2_O_2_ for 1 h. **c** The levels of hNRROS mRNA expression were determined by RT/real-time PCR. **d** The cells were labeled with 10 μM H_2_DCFDA and then incubated with 100 μM H_2_O_2_ for 30 min. The levels of ROS generation were observed using a fluorescence microscope. Scale bar = 100 μm. **e** RBA-1 stable clones of hNRROS and pCMV-Tag2B, or RBA-1 cells were treated with 100 μM H_2_O_2_ for the indicated time intervals. The levels of protein expression were analyzed by Western blotting. **f**, **g** RBA-1 stable clones were treated with H_2_O_2_ for the indicated time intervals. The levels of IL-6 mRNA expression and secretion of IL-6 were analyzed by RT/real-time PCR and ELISA kit, respectively. The data are presented as mean ± SEM, from three independent experiments (*n* = 3, number of independent cell culture preparations). *p < 0.05, as compared with the respective values significantly different as indicated

15d-PGJ_2_ has been shown to display neuroprotection against ROS stress (Abdo et al. 2012; Wu et al. 2016). We proposed that 15d-PGJ_2_ is capable of regulating Nox activity and dampens ROS generation in RBA-1 cells. The results of Western blotting showed that H_2_O_2_ time-dependently stimulated p47^phox^ phosphorylation and gp91^phox^ expression, which were attenuated by pretreatment with 15d-PGJ_2_ through upregulation of NRROS (*p* < 0.05, as compared the control, Fig. 7a). Moreover, 15d-PGJ_2_ also inhibited the H_2_O_2_-induced IL-6 mRNA expression (*p* < 0.05, as compared the control, Fig. 7b), suggesting an anti-inflammatory effect of 15d-PGJ_2_ on the H_2_O_2_-mediated responses.

**Fig. 8:**
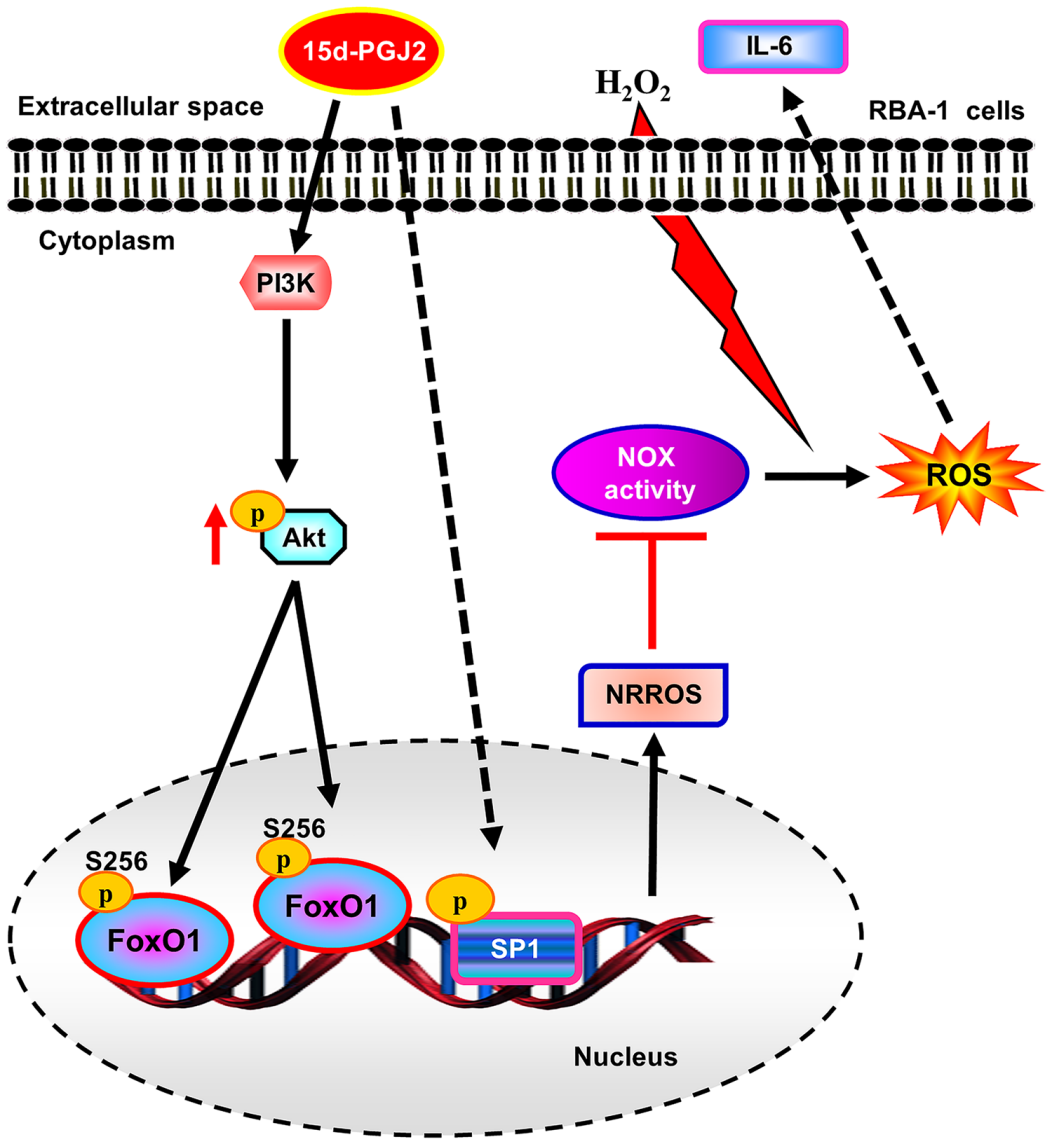
Scheme of signaling pathways is involved in 15d-PGJ_2_-induced NRROS expression suppressing the H_2_O_2_-induced IL-6 expression and ROS generation in RBA-1 cells. NRROS expression induced by 15d-PGJ_2_ is mediated through a PPARγ-independent activation of PI3K/AKT leading to phosphorylation of FoxO1 and Sp1. These activated transcription factors bind to NRROS promoters and enhance NRROS transcription in RBA-1 cells. Up-regulation of NRROS not only downregulates the H_2_O_2_-mediated ROS generation but also inhibits the expression of pro-inflammatory cytokine IL-6. These results elucidate the mechanisms underlying 15d-PGJ_2_-induced NRROS expression which might be a potential strategy for management of brain inflammatory and neurodegenerative diseases

We noticed that H_2_O_2_ regulates downstream of signaling components in various types of cells (Miller et al. 2010). To analyze whether NRROS regulated Nox activity in RBA-1 cells, full-length human NRROS was constructed into a pCMV-Tag2B vector. RBA-1 cells were transfected with pCMV-hNRROS (human NRROS) and selected with G418 to establish constitutively expressed clones. The RT/real-time PCR was performed to determine the levels of human NRROS mRNA expression in RBA-1 cells. As shown in Fig. 7c, the levels of overexpressed human NRROS mRNA and protein were detected in pCMV-hNRROS constitutively expressed RBA-1 cells. In addition, H_2_O_2_ treatment evoked the levels of ROS generation in RBA-1 cells, which was attenuated by constitutive expression of pCMV-hNRROS stable clone, using H_2_DCFDA observed under a fluorescent microscope (*p* < 0.05, as compared the control, Tag2B or RBA-1 cells, Fig. 7d). These results suggested that NRROS attenuates p47^phox^ phosphorylation and suppresses the Nox activity (gp91^phox^ expression) in H_2_O_2_-treated RBA-1 cells.

Excessive generation of ROS results in the secretion of pro-inflammatory cytokines such as TNF-α, IL-1, and IL-6 to reflect the proinflammatory responses in M1 macrophages (Mittal et al. 2014). H_2_O_2_ activates NF-κB leading to IL-6 transcription and secretion (Zhang et al. 2001), which is involved in the development of several autoimmune diseases (Kobayashi et al. 2016). To further evaluate the effect of NRROS on ROS-mediated inflammatory responses, H_2_O_2_-induced IL-6 mRNA transcription and secretion were examined in the constitutively NRROS-expressed RBA-1 cells, determined by using RT/real-time PCR and IL-6 ELISA assays, respectively. We found that H_2_O_2_ induced IL-6 mRNA expression and secretion in parental RBA-1 cells and the vector control (Tag2B) clone, which were attenuated in the constitutively NRROS-expressed RBA-1 cells (*p* < 0.05, as compared the control, Tag2B or RBA-1 cells, Fig. 7e, f). These results indicated that constitutively expressed NRROS reduces the H_2_O_2_-induced IL-6 mRNA transcription and secretion in RBA-1 cells.

## Discussion

The antioxidant enzymes, including superoxidase dismutase, catalase, glutathione peroxidase, peroxiredoxins, and thioredoxins, cooperate to remove ROS (Mittal et al. 2014). Noubade et al. identified the regulatory activity of NRROS which interferes the Nox complex formation through the interaction of NRROS-Nox-2 and the ER-dependent Nox-2 degradation in macrophages (Noubade et al. 2014). However, there was little information concerning NRROS to regulate Nox activity and ROS generation. Here, we established the essential regions of NRROS transcription and the 15d-PGJ_2_ response region on NRROS promoters. Figure 7 depicts that transcription factors Sp1 and FoxO1 response elements located on the upstream of exon 1 of rat NRROS gene and contributed to transcriptional regulation mediated through a PI3K/Akt-dependent FoxO1 pathway. In addition, 15d-PGJ_2_ activated both FoxO1 and Sp1 association with the response elements of NRROS promoter to accelerate NRROS transcription. Upregulation of NRROS attenuated the p47^phox^ phosphorylation and Nox/ROS generation stimulated by H_2_O_2_, which downregulated IL-6 expression in RBA-1 cells.

ROS are important signaling molecules in the pathogenesis of inflammatory disorders. Several sources of ROS generation, such as Nox, mitochondria, uncoupled NOS, and xanthine oxidoreductase, compose the ROS generation systems in various types of cells (Mittal et al. 2014). Nox complex generates ROS in various tissues and cells in response to several stresses (Mittal et al. 2014). The regulation of normal physiological functions and the inflammatory responses are dependent on the cellular concentrations of ROS (Kamata and Hirata 1999). Excessive ROS are a causative role in many pathologies of airway diseases (Lee and Yang 2012; Rahman et al. 2006). The stimulated immune cells initiate enzymatic activity of Nox complex to produce superoxide anion during encountering inhaled microorganisms or other mediators. H_2_O_2_ can cross cell membranes through aquaporin channels such as AQP3 and AQP8, which also mediate membrane H_2_O_2_ uptake and raising the permeability for H_2_O_2_ entering into cells (Bienert et al. 2007; Miller et al. 2010). Several reports indicate that H_2_O_2_ activates signaling pathways to enhance ROS production in different types of cells (Griendling et al. 2000; Torres and Forman 2003). We observed that constitutively expressed NRROS attenuates the H_2_O_2_–induced ROS signal. We also noticed that the role of NRROS is a negative regulatory protein to limit ROS generation. Catalase, superoxide dismutases, and glutathione peroxidase are the ROS-eliminating enzymes. We suggested that constitutively expressed NRROS not only reduces ROS generation but also cooperates with the activities of catalase and glutathione peroxidase to eliminate ROS, such as the exogenous H_2_O_2_. Our data indicated that constitutive expression of human NRROS reduced the H_2_O_2_-induced Nox/ROS generation, which protected against brain inflammatory diseases (Fig. 8).

NOX complex assembling and activity are mediated by complex formation of gp91^phox^ and phosphorylated p47^phox^. Regarding the role of the p47^phox^, many kinases are involved in the phosphorylation of p47^phox^ and it is absolutely required for Nox assembly and activation. We noticed that phosphorylation at Ser^370^ (the antigen usage of phosphorylated p47^phox^ antibody) had no significant effect on the p47^phox^ interaction with other phox subunits, which was necessary for regulation of Nox activation (El-Benna et al. 2009; Meijles et al. 2014). Several insults, such as pro-inflammatory cytokines, LPS, phorbol ester, and cellular metabolite inducers, trigger p47^phox^ phosphorylation to regulate Nox activity (Mittal et al. 2014). These stimuli initiate the signal transduction to activate downstream kinases. Protein kinase Cs, protein kinase A, Akt, ERK1/2, and p38 MAPK are involved in the regulation of Nox activity via p47^phox^ phosphorylation (El-Benna et al. 2009). The astrocytes activated by pathological stresses produce ROS to protect against microbial infection. On the other side, ROS generation also contributes to neurodegeneration. The astrocytic Nox activity plays an important role in CNS physiology and pathology (Abramov et al. 2005). Furthermore, we found that 15d-PGJ_2_ reduced the H_2_O_2_-induced p47^phox^ phosphorylation, gp91 ^phox^ expression, and IL-6 expression. We attempted to establish the relationship among NRROS expression, antioxidative stress, and anti-inflammation in RBA-1 cells. 15d-PGJ_2_ inhibited the H_2_O_2_-induced p47^phox^ phosphorylation and IL-6 mRNA expression through NRROS expression. We found that stable expression of hNRROS reduced the p47^phox^ phosphorylation and pg91^phox^ expression. On the other side, the zymosan-induced ROS generation is reduced by NRROS expression in BMDMs (Noubade et al. 2014). Our results were consistent with this report indicating that stable expression of human NRROS reduced the H_2_O_2_-induced ROS generation in RBA-1 cells. Based on these findings, we suggested that NRROS attenuates the phosphorylation of p47^phox^ and Nox-2 activity (gp91^phox^) in RBA-1 cells.

15d-PGJ_2_, an endogenous ligand of PPARγ, induces PPARγ-dependent or independent cell apoptosis in cancer cells (Ray et al. 2006; Shimada et al. 2002) and regulates the inflammatory responses (Kim et al. 2007). In the regulation of inflammatory and immune responses, 15d-PGJ_2_ blocks IKK activity and association of NF-κB to κB sites of promoter to inhibit expression of genes via a PPARγ-independent pathway (Giri et al. 2004; Straus et al. 2000). 15d-PGJ_2_ also reduced expression of several pro-inflammatory cytokines and exhibited neuronal protecting ability against CNS inflammation (Giri et al. 2004; Wu et al. 2014). Our studies suggested that both of the PPARγ-dependent and PPARγ-independent pathways are involved in the regulation of 15d-PGJ_2_-induced NRROS to attenuate H_2_O_2_-induced pro-inflammatory cytokine (IL-6) expression. In addition, we found that 15d-PGJ_2_ induced serial phosphorylation of signal molecules through PI3K/AKT/FoxO1. Pretreatment with inhibitors and gene-specific knockdown (siRNA transfection) obtained the consistent results, suggesting that 15d-PGJ_2_ triggers phosphorylation cascade through PI3K/AKT/FoxO1 in RBA-1 cells to induce NRROS expression. Furthermore, the PPARγ response element was not present on rat NRROS promoter region. However, pretreatment with GW9662, a PPARγ antagonist, reduced the 15d-PGJ_2_-induced NRROS mRNA expression. Based on these results, we suggested that cooperative effects of PPARγ-independent and PPARγ-dependent pathways were involved in the regulation of 15d-PGJ_2_-induced NRROS expression. In addition, we also noticed that PPARγ agonists trigger several signal components to regulate gene expression in different types of cells or tissues (Mix et al. 2004; Phulwani et al. 2006; Wei et al. 2014). Therefore, the role of PPARγ response element in these responses is needed for further studies.

The transcription factor FoxOs contain the FoxO consensus motif and associated ability to response element of promoter in regulation of transcriptional activation or repression. To regulate FoxO activities, posttranslational modifications affect the FoxO-mediated transcriptional activity, subcellular localization, and protein stability (Fu and Tindall 2008). Previous reports indicate that FoxO1 affects cellular responses including metabolism, differentiation, and apoptosis by the Akt-dependent phosphorylation at Ser^256^ of FoxO1 (Fu and Tindall 2008; Savai et al. 2014). Pretreatment with inhibitor, AS1842856, reduced the phosphorylation of FoxO1. In this report, we found that pretreatment with respective inhibitors or transfection with siRNA of PI3K, Akt, or FoxO1 attenuated the 15d-PGJ_2_-induced NRROS expression in RBA-1 cells. The phosphorylation of these signal molecules indicated the similar results that PI3K/Akt-phosphorylated Ser^256^ of FoxO1 was involved in the 15d-PGJ_2_-mediated responses. This PPARγ-independent pathway may be mediated through the TATA-less promoter activity of NRROS. We noticed that 15d-PGJ_2_-stimulated phosphorylated FoxO1 (Ser^256^) binding to the promoter was blocked by a FoxO1 inhibitor, but not by a Sp1 inhibitor (Supplementary Fig. S1a) on the Sp1 binding region. On the FoxO1-binding regions, both phosphorylated FoxO1 and Sp1 bindings were attenuated by AS1842856 and mithramycin A (Supplementary Fig. S1b). The results of ChIP assay suggested that the phosphorylated FoxO1 association on FoxO1-binding regions are cooperated with Sp1 to accelerate NRROS transcription in RBA-1 cells treated with 15d-PGJ_2_. In addition to FoxO1 and Sp1, Nrf2 has been known to be a master transcription factor for up-regulation of antioxidant proteins including heme oxygenase-1 in various types of cells (Haskew-Layton et al. 2013; Lin et al. 2018; Shih et al. 2003). In this study, we also found that 15d-PGJ_2_ time-dependently stimulated Nrf2 phosphorylation which was attenuated by transfection with Nrf2 siRNA which knocked down the level of Nrf2 protein in RBA-1 cells (Supplementary Fig. 3a). Because transfection with Nrf2 siRNA knocked down the level of Nrf2 protein and could be applied to evaluate its role in the 15d-PGJ_2_-mediated responses. Thus, the role of Nrf2 in NRROS expression was further investigated by transfection with Nrf2 siRNA which downregulated the level of Nrf2 protein and subsequently attenuated the 15d-PGJ_2_-induced NRROS expression (Fig. 6a). These results suggested that Nrf2 also plays an important role in the NRROS expression induced by 15d-PGJ_2_.

Previous reports also indicated that Akt phosphorylates FoxO1 at Ser^256^ and promotes the association of 14-3-3 and Foxo1 degradation. The posttranslational modification of FoxO1 induces gene expression to regulate cellular functions in several types of cells (Tzivion et al. 2011; Zhang et al. 2011). FoxOs could mediate ROS detoxification through upregulation of catalase or MnSOD to reduce the oxidative stress (Akasaki et al. 2014; Zhang et al. 2011). Based on our results, we found the novel characters in Akt-mediated FoxO1 phosphorylation in 15d-PGJ_2_-treated RBA-1 cells, confirmed by transfection with either Akt or FoxO1 siRNA which downregulated the NRROS induction. We also noted that 15d-PGJ_2_-stimulated Akt phosphorylation may be due to the covalent modification of cysteine residues on phosphatase and tensin homolog and inactivation of its activity (Suh et al. 2018). This is an important issue for further study in the future. Moreover, the results of ChIP assay indicated that 15d-PGJ_2_-induced Ser^256^ phosphorylation of FoxO1 bound on NRROS promoter. The Western blotting of subcellular fraction also demonstrated that abundant Ser^256^ phosphorylation of FoxO1 was detected in the nuclear fraction. Overexpression of the phosphorylation-mimic mutant, S256D-FoxO1, also upregulated the NRROS expression. These results suggested that 15d-PGJ_2_-induced NRROS expression reduces the ROS stress through Akt-dependent FoxO1 phosphorylation in RBA-1 cells. Further, 15d-PGJ_2_ exerts neuroprotection from oxidative stress in astrocytes (Haskew-Layton et al. 2013). These protecting effects of 15d-PGJ_2_ are mediated through upregulation of Nrf2-dependent antioxidant proteins (Itoh et al. 2004). In this study, we found that Nrf2 played a key role in the NRROS expression induced by 15d-PGJ_2_ through its phosphorylation. The inductive effect of 15d-PGJ_2_ on NRROS expression was blocked by its respective antibody. Therefore, Nrf2 also plays an important in the 15d-PGJ_2_-mediated responses in RBA-1 cells.

Our findings proposed evidence to support the anti-inflammatory ability of 15d-PGJ_2_ in brain astrocytes. 15d-PGJ_2_ has been shown to reduce the expression of pro-inflammatory cytokines (such as IL-1β, IL-6, and TNF-α) and proteins (such as iNOS, COX-2, and ICAM-1) (Giri et al. 2004; Jiang et al. 1998; Ricote et al. 1998). Astrocyte-derived cytokines and chemokines also play important roles in neuroprotection or neurotoxicity in brain lesions and neurological diseases (Choi et al. 2014). Especially, we focused on the ROS-mediated expression of astrocyte-secreted cytokines such as IL-6. The elevated IL-6 by brain injury or inflammation is detected in the cerebrospinal fluid of patients with several neuronal diseases (Van Wagoner and Benveniste 1999; Van Wagoner et al. 1999). The IL-6 is a typical marker for inflammatory and immunological reaction (Kobayashi et al. 2016). Previous report indicates that IL-6 has beneficial potential by neurotrophic and neuroprotective effects in CNS or accelerates the pathophysiological responses in CNS disorders (Van Wagoner et al. 1999). The oxidative stresses induce IL-6 expression in several types of cells (Giri et al. 2004; Zhang et al. 2001). We obtained the similar results in RBA-1 cells showing that H_2_O_2_ induced IL-6 mRNA and protein expression. The constitutive expression of human NRROS inhibited p47^phox^ phosphorylation, gp91^phox^ expression, and attenuated ROS generation and IL-6 mRNA expression. These results suggested that NRROS plays a regulatory role in ROS-dependent IL-6 expression and astrocyte reaction.

In summary, we found that 15d-PGJ_2_-induced NRROS expression is mediated through a PPAR-γ-independent pathway and established in the essential regions of the transcriptional initiation and 15d-PGJ_2_ response element in RBA-1 cells. 15d-PGJ_2_ stimulated FoxO1 phosphorylation mediated through PI3K/Akt pathway and activated Sp1 to regulate the NRROS transcription. In addition, Nrf2 played a key role in NRROS expression induced by 15d-PGJ_2_ which was mediated through its phosphorylation. Upregulation of NRROS attenuated the p47^phox^ phosphorylation and suppressed ROS generation leading to a decrease in the expression of IL-6. Based on these findings, we found the first time that up-regulation of NRROS by 15d-PGJ_2_ provides useful therapeutic strategies for brain injury, inflammation, and neurodegenerative diseases.

## Supplementary Information

Below is the link to the electronic supplementary material.

Supplementary file1 (TIF 200 KB)

Supplementary file2 (TIF 233 KB)

## Abbreviations

BMDMs: Bone marrow-derived macrophages
ChIP: Chromatin immunoprecipitation
CNS: Central nervous system
15d-PGJ_2_: 15-Deoxy-∆12,14-prostaglandin J_2_
ECL: Enhanced chemiluminescence
ELISA: Enzyme-linked immunosorbent assay
FBS: Fetal bovine serum
GFAP: Glial fibrillary acid protein
GPx: Glutathione peroxidase
IFN: Interferon
IL: Interleukin
Nox: NADPH oxidase
NRROS: Negative regulator of ROS
PPAR: Peroxisome proliferator-activated receptor
RBA-1: Rat brain astrocytes
ROS: Reactive oxygen species
SOD: Superoxidase dismutase
TBP: TATA-binding protein
TFIIB: Transcription factor IIB
Trxs: Thioredoxins
