# Supplementary figures and images for: Involvement of FoxO1, Sp1, and Nrf2 in Upregulation of Negative Regulator of ROS by 15d-PGJ2 Attenuates H2O2-Induced IL-6 Expression in Rat Brain Astrocytes
Source: Neurotox Res. 2022 Jan 8;40(1):154–72. doi: 10.1007/s12640-020-00318-6 (PMC8784370; doi:10.1007/s12640-020-00318-6)

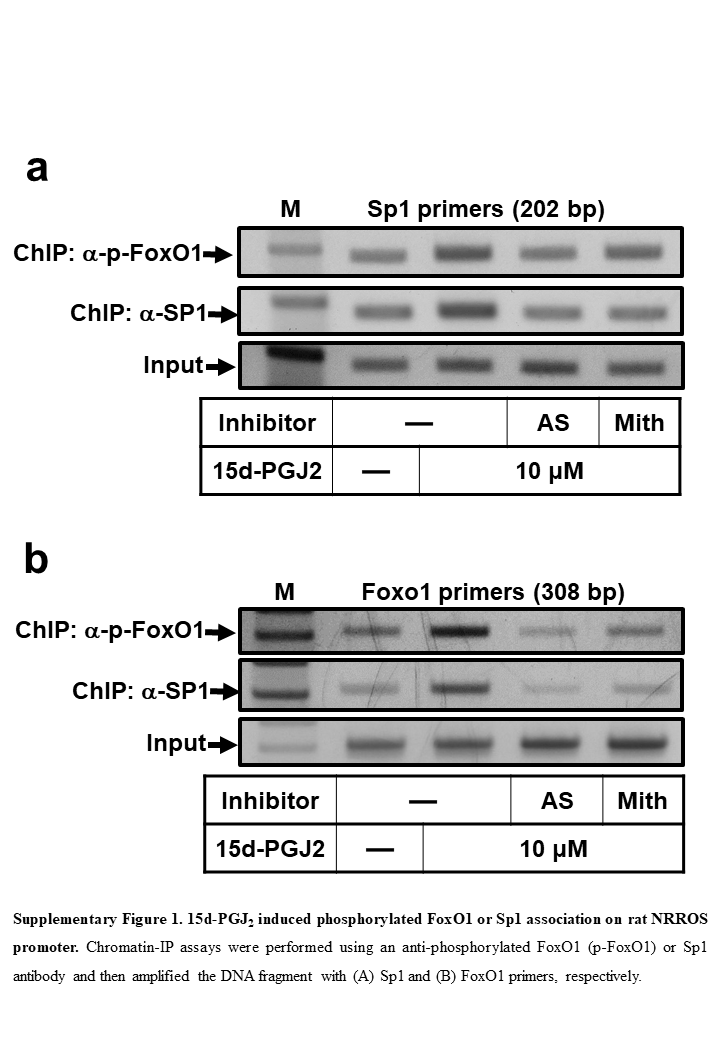

Supplement: Supplementary file 1 — Supplementary file1 (TIF 200 KB) [file 12640_2020_318_MOESM1_ESM.tif]

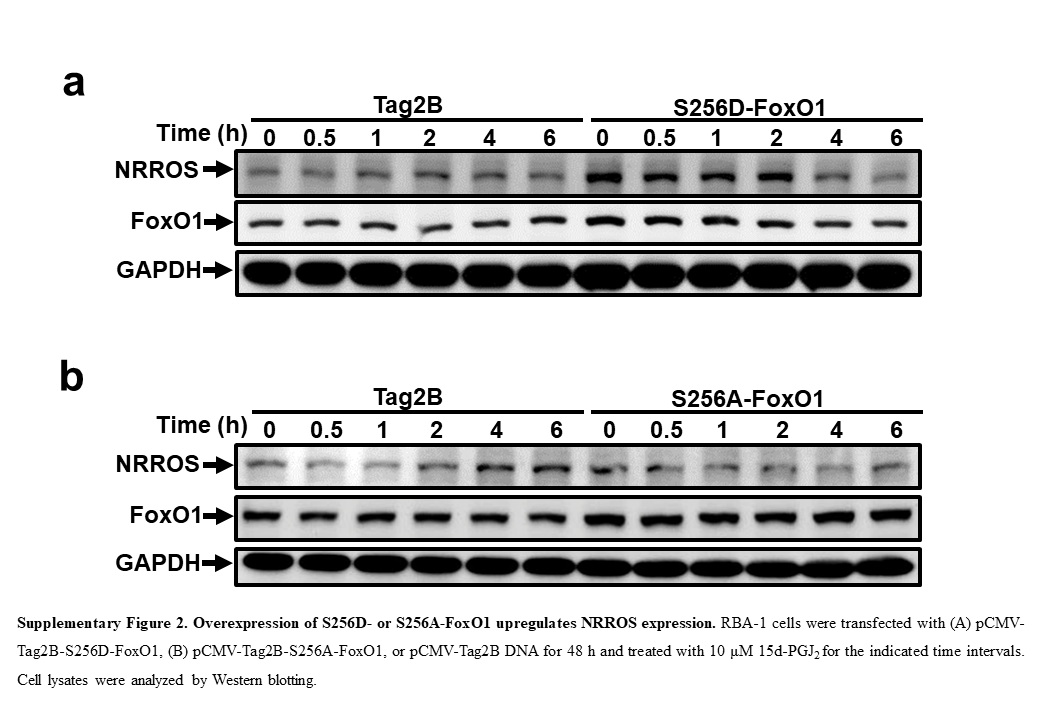

Supplement: Supplementary file 2 — Supplementary file2 (TIF 233 KB) [file 12640_2020_318_MOESM2_ESM.tif]
